# The essential role of gut microbiota in dsRNA-mediated pest control of the phytophagous ladybird beetle, *Henosepilachna vigintioctopunctata*

**DOI:** 10.1038/s41522-025-00767-x

**Published:** 2025-07-22

**Authors:** Yujie Huang, Jinman Huang, Dae Sung Kim, Jiang Zhang

**Affiliations:** 1 School of Life Sciences, Hubei University, Hubei Hongshan Laboratory, Wuhan, China; 2 Shenzhen Branch, Guangdong Laboratory of Lingnan Modern Agriculture, Key Laboratory of Synthetic Biology, Ministry of Agriculture and Rural Affairs, Agricultural Genomics Institute at Shenzhen, Chinese Academy of Agricultural Sciences, Shenzhen, China

**Keywords:** Biological techniques, Microbiology

## Abstract

RNA interference (RNAi) has proven to be an effective measure in combating insect pests. RNA-based pesticides have already made their way to the market for pest control. We previously have demonstrated the pivotal role of gut microbiota in influencing the efficacy of RNAi against the leaf beetle *Plagiodera versicolora* (Coleoptera), a species highly susceptible to RNAi. However, the role of the gut microbiota in different insect species remains ambiguous. We delved into the intricate interplay between gut bacterial communities and RNAi in the phytophagous ladybird beetle, *Henosepilachna vigintioctopunctata* (HV), a significant agricultural pest targeting solanaceous plants. By administering double-stranded RNA targeting HV *β-Actin* gene (ds*ACT*) or utilizing transplastomic (plastid genome transformed) potato plants expressing ds*ACT* on HV axenic larvae, a substantial decrease in lethality was observed compared to non-axenic controls. This underscores the critical role of microbiota in enhancing the effectiveness of RNAi. The disruption of microbial diversity and functionality due to dysbiosis induced by dsRNA feeding amplified physiological stress and mortality rates in HV. Notably, the optimal bacterial densities were crucial for maximizing mortality rates, as elevated concentrations of gut bacteria could disrupt the native microbial equilibrium. These findings underscore the importance of microbiota-aware strategies in RNAi applications, particularly in the context of sustainable pest management within solanaceous crop systems.

## Introduction

Traditional pest management methods heavily reliant on chemical pesticides face numerous challenges including pest resistance development, environmental contamination, and harm to non-target organisms^1^. These issues underscore the pressing need for novel, sustainable, and precisely targeted pest control approaches. RNA interference (RNAi) has emerged as a promising solution for pest management due to its specificity and reduced environmental impact. This mechanism involves the silencing of specific genes through the introduction of double-stranded RNA (dsRNA), which triggers the degradation of target mRNA, effectively suppressing gene expression^2^. In agriculture, dsRNA can be tailored to target crucial genes in pest species, leading to their mortality or decreased fitness^3,4^. This innovative technique provides a highly precise means to disrupt pest biology without the broad ecological disturbances associated with conventional chemical pesticides. RNAi has demonstrated significant potential in controlling various insect species, particularly coleopterans such as the Colorado potato beetle, the western corn rootworm, and the 28-spotted ladybird beetle, by targeting essential pest genes^4–6^. The specificity of RNAi permits the targeting of pest species with minimal impact on beneficial insects and other non-target organisms^7^. Nonetheless, the efficacy of RNAi-based pest control strategies can vary substantially across different insect species, underscoring the importance of comprehending the complex biological and environmental factors influencing RNAi effectiveness.

One of the emerging factors capturing scientific interest is the intricate role of gut microbiota in modulating the insecticidal effects of dsRNA^8,9^. The gut microbiota, a complex community of microorganisms residing in the digestive tracts of insects, plays a crucial role in various physiological functions^10,11^. These functions encompass aiding in nutrient digestion, detoxifying harmful compounds, and conferring protection against pathogens^12^. Recent research has unveiled that gut bacteria are not mere bystanders in pest biology but active contributors to the outcomes of RNAi. For example, in the willow leaf beetle (*Plagiodera versicolora*), investigations have demonstrated that gut bacteria enhance the efficacy of dsRNA by transitioning from a commensal to a pathogenic status. This transition, often induced by dsRNA-triggered dysbiosis, establishes a hostile internal environment in the insect, hastening mortality rates^8^. These discoveries emphasize the necessity to delve into the intricate interplay between gut microbiota and RNAi across various pest species to refine and enhance pest control strategies.

An intriguing advancement in the realm of pest management involves the utilization of transplastomic plants to deliver dsRNA to insect pests. Plastid-mediated RNAi (PM-RNAi) presents numerous benefits, such as the stability and robust expression of dsRNA within the plastids of host plants^4,6,13,14^. This strategy has proven successful in targeting a diverse array of insect pests, with recent research showcasing its heightened efficacy when coupled with the gut microbiota’s involvement in dsRNA degradation and dysbiosis^9^. For instance, the implementation of PM-RNAi in transplastomic poplar plants engineered to express dsRNA against *P. versicolora* resulted in notably increased mortality rates in non-axenic larvae compared to axenic counterparts. This outcome highlights the synergistic impact of gut bacteria and dsRNA in perturbing pest physiology. Likewise, the incorporation of gut bacteria dynamics into the mode of action of *Bacillus thuringiensis* (Bt) crystal proteins has shed light on how the gut microbiota contributes to augmenting insecticidal activity^15^.

Within agricultural ecosystems, *Henosepilachna vigintioctopunctata*, commonly referred to as the 28-spotted ladybird beetle, stands out as a notorious pest insect. This beetle poses a significant threat to solanaceous crops like potatoes, tomatoes, and eggplants, inflicting substantial defoliation and yield losses^16,17^. Both the larval and adult stages of *H. vigintioctopunctata* exhibit highly destructive feeding behaviours, rendering it a major menace to crop productivity, particularly in regions where solanaceous crop are extensively cultivated^16^. Despite its close evolutionary relationship to beneficial ladybird beetles, the pestilent nature of *H. vigintioctopunctata* necessitates the development of effective species-specific management strategies^17^. Given the pestiferous impact of *H. vigintioctopunctata*, comprehending the factors influencing RNAi sensitivity in this species is imperative for formulating targeted and efficient pest control measures^6^. Moreover, considering the crucial role of gut microbiota in modulating RNAi efficacy observed in other insect species^8,9^, exploring this relationship in *H. vigintioctopunctata* could yield invaluable insights into microbiota-informed RNAi approaches.

This study endeavours to illuminate the pivotal role of gut microbiota in modulating RNAi sensitivity in *H. vigintioctopunctata*. By delving into the intricate interplay between gut microbial communities and dsRNA in this economically significant pest, our aim is to bolster the efficacy of RNAi-based pest control strategies. Through the generation of axenic larvae, we have uncovered the indispensable contribution of gut microbiota in amplifying the lethal impact of ds*ACT* feeding on *H. vigintioctopunctata*. Our investigation reveals that dsRNA ingestion induces dysbiosis within the gut microbiota, leading to notable alterations in microbial composition and diversity. These functional changes could exacerbate the physiological stress experienced by the larvae, thereby intensifying RNAi-mediated lethality. All tested bacterial strains have been shown to enhance RNAi efficacy by influencing ds*ACT*-induced larval mortality in a dosage-dependent manner, underscoring the necessity of optimal bacterial densities to optimize RNAi-induced lethality. Interestingly, while this heightened mortality effect was observed in axenic larvae, non-axenic larvae remained unaffected, suggesting that the existing level of native microbiota is adequate to magnify the lethal effects of ds*ACT*. Furthermore, we have demonstrated the indispensable role of gut microbiota in managing *H. vigintioctopunctata* concerning dsRNA-expressing transplastomic potatoes. These findings hold promise for informing the development of tailored, sustainable, and microbiota-informed pest management solutions geared towards safeguarding solanaceous crops.

## Results

### Confirmation of axenic and non-axenic larvae

**Fig. 1:**
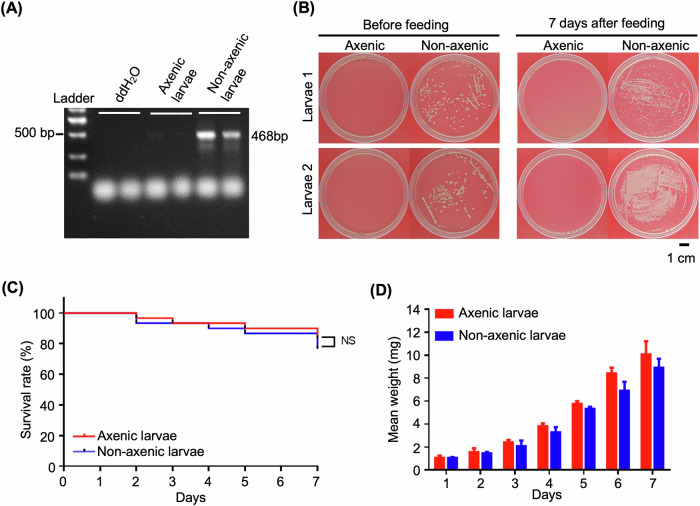
Confirmation of the axenic status of *Henosepilachna vigintioctopunctata* larvae and comparison of axenic and non-axenic groups before and after feeding. **A** DNA extraction from axenic and non-axenic larvae followed by PCR amplification using a primer pair targeting 16S *rRNA* to confirm the axenic status. **B** Assessment of bacterial colony formation on LB agar plates (*n* = 5, gut extracts were diluted 10^4^-fold with sterilized H_2_O) before and 7 days after feeding axenic and non-axenic *H. vigintioctopunctata* larvae with aseptic *S. nigrum* leaves. **C** Kaplan–Meier survival curves of axenic and non-axenic *H. vigintioctopunctata* larvae fed on aseptic *S. nigrum* leaves for seven days (*n* = 30). The log-rank test was utilized to assess the significance of differences between the two groups. NS not significant. **D** Comparison of the weight of axenic and non-axenic *H. vigintioctopunctata* larvae fed on aseptic *S. nigrum* leaves for seven days.

To evaluate the impact of gut microbiota on RNAi effectiveness in *H. vigintioctopunctata*, we initially established and verified axenic and non-axenic larvae. PCR amplification targeting the 16S *rRNA* gene revealed that axenic larvae exhibited no detectable bacterial DNA, contrasting with non-axenic larvae which displayed a robust 468 bp amplification product, signifying the presence of a gut microbiota community (Fig. 1A). This molecular evidence was supported by microbiological assays: LB agar inoculated with extracts from axenic larvae showed no bacterial colonies, while non-axenic larvae consistently exhibited dense bacterial growth (Fig. 1B). Survival and growth assessments demonstrated no significant variances between axenic and non-axenic larvae when fed sterile *S. nigrum* leaves for seven days. Kaplan–Meier survival analysis indicated comparable survival rates in both groups, suggesting that the absence of gut microbiota did not impact larval viability under sterile feeding conditions (Fig. 1C). Likewise, the larval weight gain during the feeding period showed similarities between axenic and non-axenic larvae (Fig. 1D). These results affirm the successful establishment of axenic *H. vigintioctopunctata* larvae and underscore that gut microbiota is not imperative for survival or growth in nutrient-rich, aseptic environments.

### Differential impact of dsRNA feeding on axenic and non-axenic larvae

**Fig. 2:**
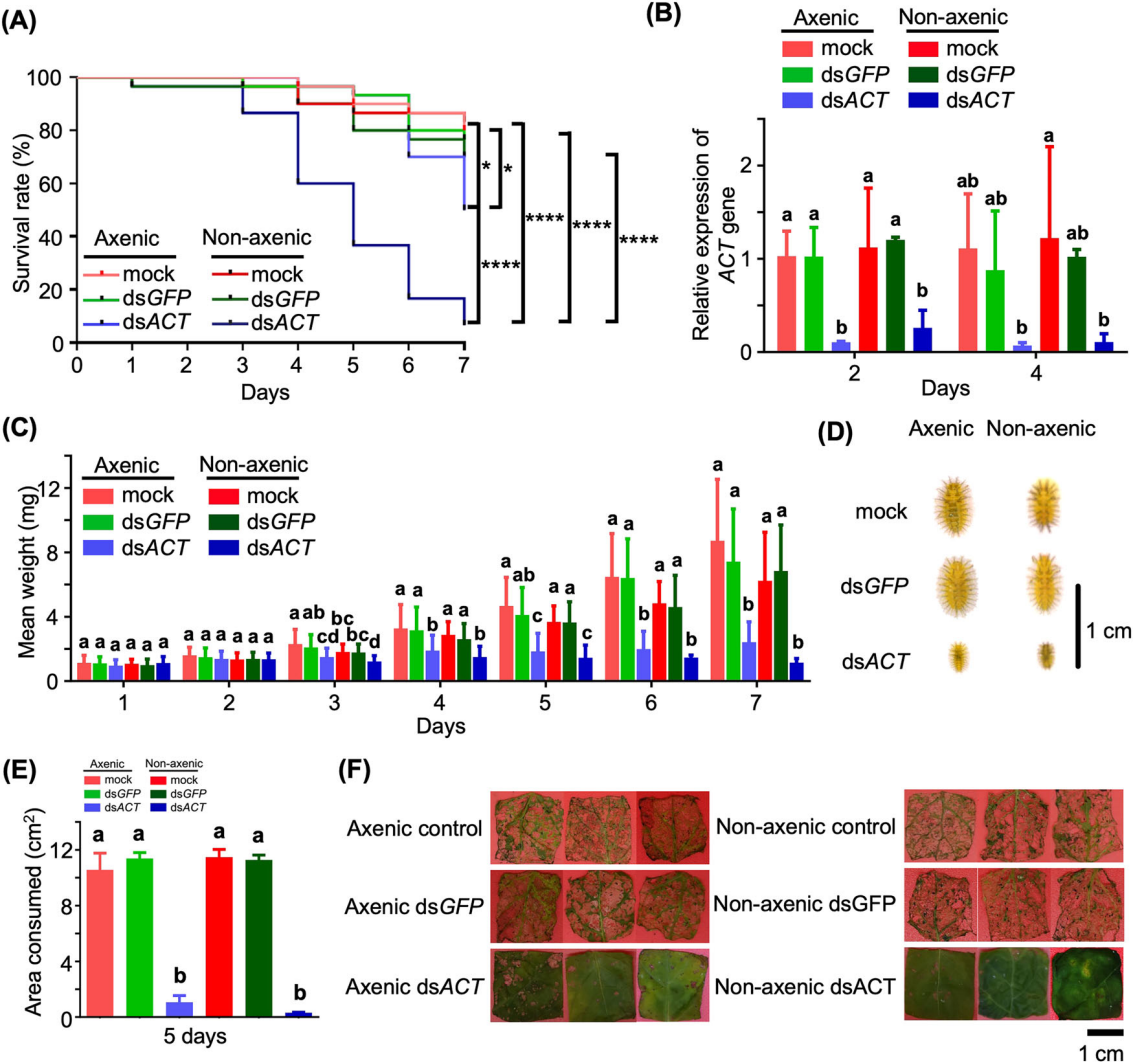
Feeding assays of *H. vigintioctopunctata* with in vitro synthesized dsRNAs. **A** Kaplan–Meier survival curves of second-instar *H. vigintioctopunctata* larvae fed with *S. nigrum* leaves painted with 4 ng/cm^2^ dsRNA (ds*ACT* or ds*GFP*) or sterilized water for axenic larvae and non-axenic larvae (*n* = 30). Sterilized water or ds*GFP* served as controls. Significance of differences between survival curves was evaluated using a log-rank test. Statistical significance indicated by asterisks (*, *P* < 0.05; ****, *P* < 0.0001). **B** Relative expression levels of *β*-*Actin* gene in *H. vigintioctopunctata* larvae from (**A**) on days 2 and 4. Gene expression levels in larvae fed with H_2_O-painted leaves set as 1. *HvRPL13* used as an internal control. Data are means ± SD (*n* = 3). Different letters above bars denote significant differences, determined by one-way ANOVA (*P* < 0.05). **C** Mean weights of surviving *H. vigintioctopunctata* larvae. Different letters above bars indicate significant differences between groups (*P* < 0.05, one-way ANOVA with Tukey’s multiple comparison test). **D** Phenotypic impact of *H. vigintioctopunctata* larvae feeding with ds*ACT* group compared to control after 7 days of feeding. Scale bar, 1 cm. **E** Comparison of feeding damage caused by *H. vigintioctopunctata* larvae after 5 days of feeding. **F** Leaf area consumed by *H. vigintioctopunctata* larvae as shown in (**E**). Scale bar, 1 cm.

The role of gut microbiota in RNAi-mediated gene silencing was investigated by feeding axenic and non-axenic larvae *S. nigrum* leaves coated with in vitro synthesized ds*ACT* or ds*GFP* (control). To evaluate RNAi efficacy, a dsRNA specific to the *β*-*Actin* gene of *H. vigintioctopunctata* was synthesized and validated. The ds*ACT* construct was designed to target a 200 bp coding sequence within the *β*-*Actin* gene (Supplementary Fig. 1A). High-quality dsRNA In vitro synthesis produced, as confirmed by agarose gel electrophoresis, displaying bands corresponding to ds*ACT* and the ds*GFP* control (Supplementary Fig. 1B). These constructs were subsequently used in RNAi feeding experiments. Kaplan–Meier survival analysis revealed dramatically distinct outcomes in the response of ds*ACT* between the two groups. Axenic larvae fed ds*ACT* exhibited near a 30% increase in mortality compared to those fed ds*GFP* (control) with a significance level of *P* < 0.05. In contrast, non-axenic larvae fed ds*ACT* experienced significant mortality, with 80% of larvae perishing by day 5, in stark contrast to minimal mortality observed in ds*GFP*-fed controls (*P* < 0.0001, Fig. 2A). These findings suggest that gut microbiota plays a crucial role in augmenting the lethality induced by ds*ACT*.

To assess RNAi efficacy, reverse transcription quantitative real-time PCR (RT-qPCR) was employed to measure the expression levels of the target *β*-*Actin* gene. *β*-*Actin* gene relative quantity was calculated using *H. vigintioctopunctata* RPL 13 (*HvRPL13*) gene as an internal control. Both axenic and non-axenic larvae fed ds*ACT* displayed substantial downregulation of *β*-*Actin* gene compared to ds*GFP*-fed controls (*P* < 0.05; Fig. 2B), with no significant differences observed between ds*ACT*-fed axenic and non-axenic groups. To assess the stability of our *β*-*Actin* gene expression data, we also determined relative *β*-*Actin* gene expression using another reference gene, *HvGAPDH*, showing a consistent result with Fig. 2B (Supplementary Fig. 2). Apart from the increased mortality in ds*ACT*-fed non-axenic larvae in comparison to ds*ACT*-fed axenic larvae, other parameters reflecting phenotypic differences between these groups were similar. Both axenic or non-axenic larvae treated with ds*ACT* exhibited significantly reduced body size and weight compared to their respective ds*GFP*-fed controls, while no significant differences were noted between axenic and non-axenic larvae fed ds*ACT* (Fig. 2C, D). Furthermore, the damaged areas caused by ds*ACT*-fed larvae were markedly decreased compared to mock controls or ds*GFP*-fed larvae, as evidenced by the reduced leaf areas consumed by ds*ACT*-fed larvae in comparison to controls (Fig. 2E, F). These results suggest that gut microbiota do not directly influence the efficiency of *β*-*Actin* gene silencing, nor do they significantly alter feeding behavior or growth during ds*ACT* treatment.

To further validate the broader implications of our findings, we targeted an additional essential gene in *H. vigintioctopunctata*–the *Soluble NSF Attachment Protein* (*SNAP*) gene–using oral dsRNA delivery. Kaplan–Meier survival analysis revealed striking differences between axenic and non-axenic larvae upon ds*SNAP* treatment. Non-axenic larvae showed a rapid decline in survival, with mortality surpassing 80% by day 5. In contrast, axenic larvae maintained ~50% survival through day 7 (Supplementary Fig. 3). These findings reinforce the role of gut microbiota in broadly enhancing RNAi efficacy across multiple lethal gene targets.

### Gut microbiota dysbiosis induced by dsRNA feeding

**Fig. 3:**
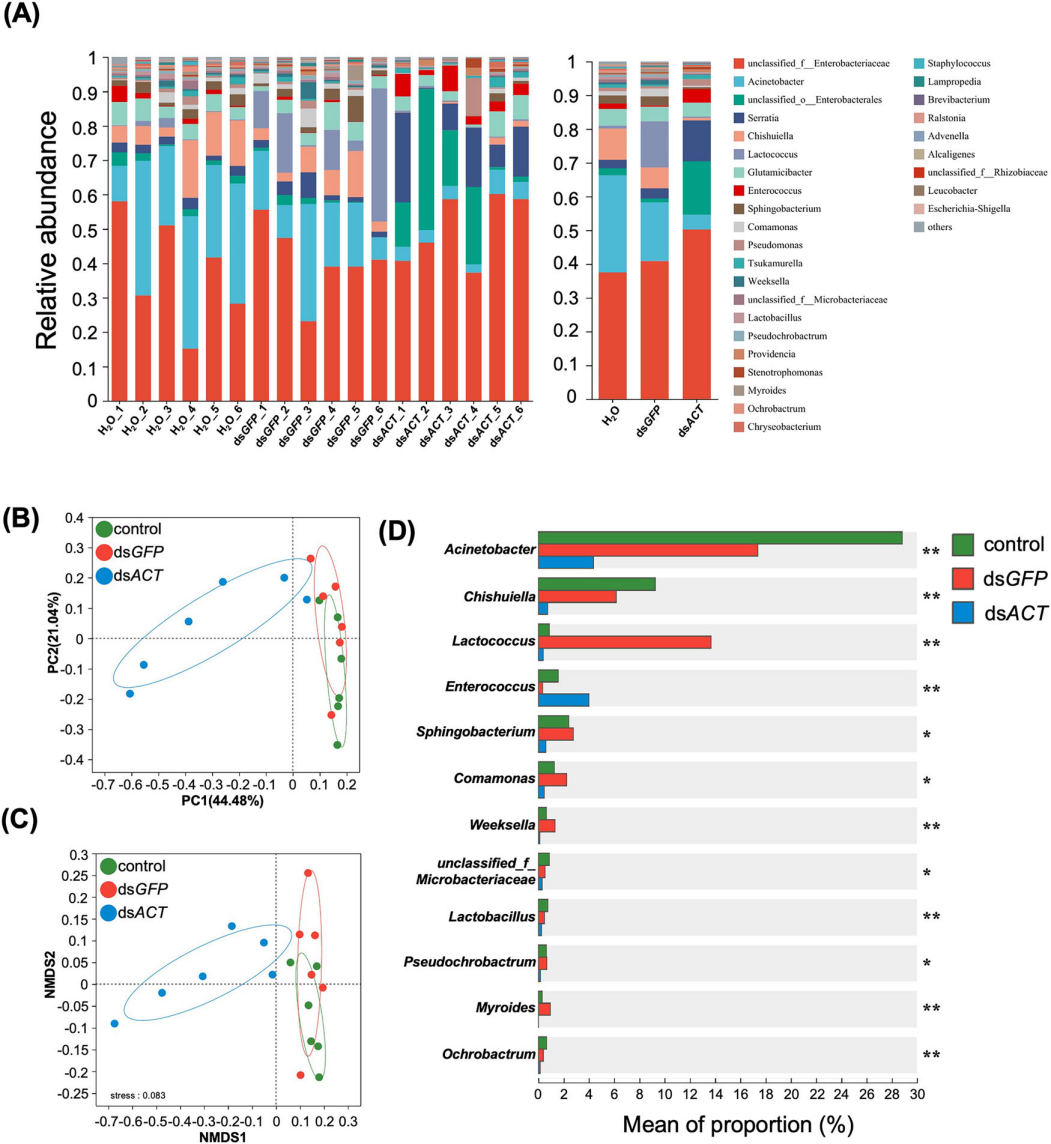
Ingestion of dsRNA induces gut microbiota dysbiosis in *H.vigintioctopunctata.* **A** Relative abundance of different genera within the gut microbiota community. The left plot displays the relative abundance from each sample, while the right plot showcases the average values of each group. **B** Principal coordinates analysis (PCoA) of 18 samples based on amplicon sequence variants (ASV) using Bray–Curtis dissimilarity to analyze bacterial community diversity. **C** Non-metric multidimensional scaling (NMDS) with ASV based on Jaccard matrix. The stress value, indicating the fit of the ordination, is presented in the bottom left of the box. **D** Bar plot analysis of Kruskal–Wallis H test for the relative proportions of 12 major genera of gut bacteria. Relative abundances of identified microbial taxa were assessed in gut samples collected from larvae fed with ds*GFP*, ds*ACT*, or H_2_O-painted leaves. *P* values were computed using the Kruskal–Wallis H test. *** *P* < 0.001; ** *P* < 0.01; * *P* < 0.05. Taxa unidentified at the genus level are indicated at the family (f) or order (o) level.

To explore the impact of dsRNA feeding on gut microbiota, 16S *rRNA* gene sequencing was conducted on gut samples collected from larvae fed leaves coated with ds*ACT*, ds*GFP*, or sterile water. The sequencing methodology for these analyses is depicted in Supplementary Fig. 4. The findings revealed significant alterations in the gut microbial community structure subsequent to ds*ACT* treatment. Key genera such as *Enterococcus* were notably enriched in ds*ACT*-fed larvae, while *Lactococcus*, *Weeksella*, and *Myroides* demonstrated significant enrichment in ds*GFP*-fed larvae. Conversely, genera like *Acinetobacter* and *Pseudochrobactrum* were diminished in the ds*ACT*-treated group compared to controls, encompassing water-treated or ds*GFP*-fed larvae (Fig. 3A, D).

Diversity assessments utilizing Principal Coordinate Analysis (PCoA) and Non-Metric Multidimensional Scaling (NMDS) revealed distinct clustering of gut microbiota from ds*ACT*-fed larvae in comparison to water controls and ds*GFP*-fed larvae (Fig. 3B, C). These results suggest that ds*ACT* feeding triggers dysbiosis in the gut microbiota, characterized by notable shifts in microbial composition and diversity. Such dysbiosis likely plays a pivotal role in amplifying RNAi effects, as the altered microbial community may facilitate the degradation of dsRNA or generate metabolites that interact with the host to increase susceptibility.

### Functional insights into gut microbiota dysbiosis

**Fig. 4:**
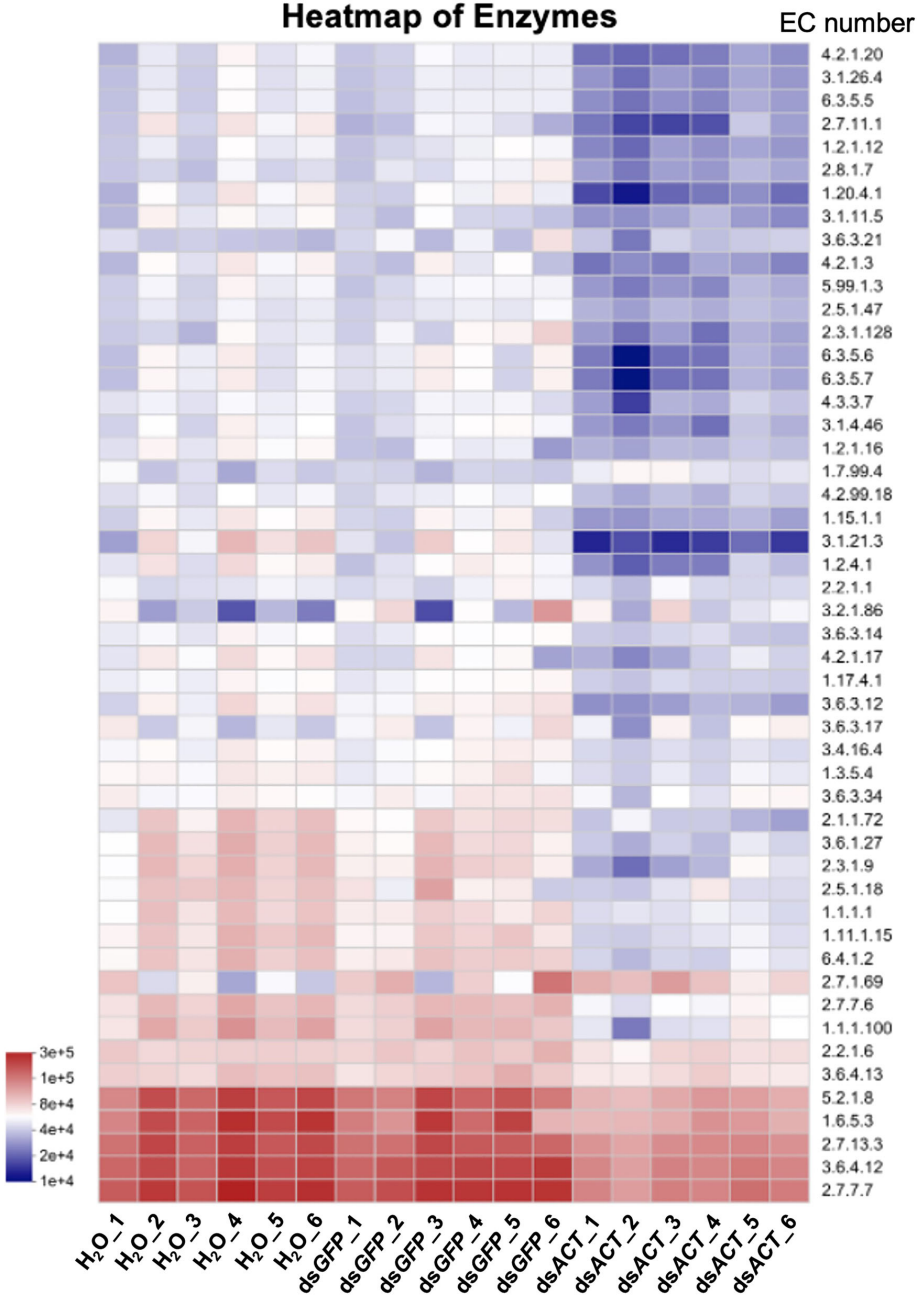
Heatmap illustrating the predicted functional enzymes by PICRUSt2 to provide insight into microbial communities in environmental samples. The heatmap displays the distribution of functional abundance and predominant functions across various samples, with each enzyme commission (EC) number indicated on the right side. The annotated names of the enzymes can be referenced in Supplementary Table 1.

Functional predictions of the gut microbiota using PICRUSt2 provided further insights into the consequences of dsRNA feeding on microbial community functions. Heatmap analysis of enzyme commission (EC) numbers highlighted the depletion or enrichment of pathways linked to amino acid and nucleotide biosynthesis in ds*ACT*-fed larvae compared to controls (Fig. 4; Supplementary Table 1). These alterations in metabolic pathways suggest that gut microbiota in ds*ACT*-fed larvae may respond to dsRNA-induced changes in the gut environment, potentially modulating their functional activity to enhance survival and proliferation under stress conditions. These functional adaptations might exacerbate the physiological stress experienced by the larvae, thereby intensifying RNAi-mediated lethality.

### Isolation of gut bacteria and its impact on larval growth

**Fig. 5:**
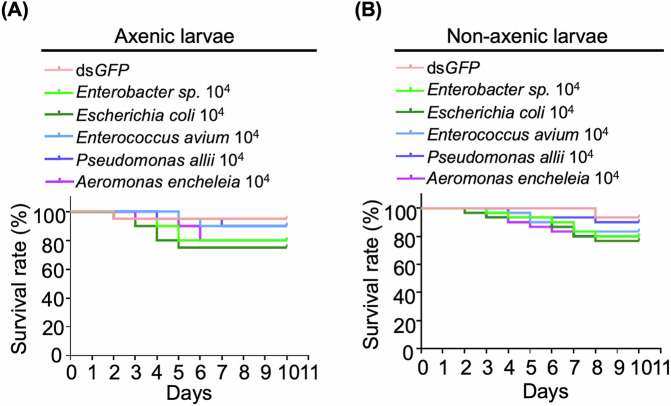
Effect of bacterial species from gut microbiota on second-instar axenic or non-axenic of *H. vigintioctopunctata* larvae. Kaplan–Meier survival curves of axenic *H. vigintioctopunctata* larvae (*n* = 30) (**A**) or non-axenic larvae (**B**) fed with different bacterial species. Bacterial density of 10^4^ cells/cm^2^ was applied to *S. nigrum* leaves.

To assess the impact of gut bacterial species on dsRNA-induced mortality, individual gut bacterial species were isolated from the second-instar non-axenic larvae group. Among these isolates, five different bacterial strains (*Enterobacter sp*., *Enterococcus avium*, *Escherichia coli*, *Pseudomonas allii*, and *Aeromonas encheleia*) representing four different taxa (Enterobacteriaceae, Enterococcaceae, Pseudomonas and Aeromonadaceae) were selected to investigate the specific contributions of bacterial species to RNAi efficacy (Supplementary Table 2). Notably, *E. avium* is the sole gram-positive bacterium among the tested strains, with the remaining four strains being gram-negative. Each isolate was reintroduced into axenic larvae. To confirm whether those bacterial isolates affect larval growth under standard conditions, each strain was applied to *S. nigrum* leaves and fed to second-instar axenic or non-axenic larvae. Kaplan–Meier survival analysis showed that all tested strains had no significant impact on larval growth compared to the ds*GFP*-fed larvae group (Fig. 5A, B), suggesting that none of the strains exhibited any pesticide-like effects. Consequently, these five bacterial strains were used for further investigations to assess whether gut microbiota-derived bacterial strains influenced ds*ACT*-mediated larval mortality.

### Enhancing mortality of re-introduction of gut bacteria into axenic larvae with lethal dsRNA

**Fig. 6:**
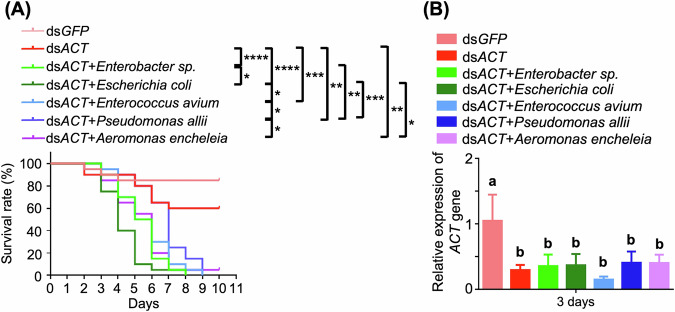
Enhancing mortality in axenic second-instar larvae with ds*ACT* feeding after reintroduction of bacterial species (*Enterobacter sp*., *E. coli*, *E. avium*, *P. allii*, *A. encheleia*). **A** Kaplan–Meier survival curves of axenic *H. vigintioctopunctata* larvae (*n* = 30) fed with ds*ACT* (4 ng / cm^2^) following the reintroduction of bacterial species. Bacterial density of 10^4^ cells/cm^2^ was applied to *S. nigrum* leaves. The significance of differences between the survival curves was evaluated using the log-rank test. ****P < 0.0001; ***P < 0.001; **P < 0.01; *P < 0.05. **B** Relative expression of the *β*-*Actin* gene in *H. vigintioctopunctata* larvae from (**A**) at 3 days post-feeding. *HvRPL13* was employed as an internal control, with gene expression levels normalized to 1 in larvae fed with ds*GFP* leaves. Data are means ± SD (*n* = 3). Different letters above bars indicate significant difference, determined through one-way ANOVA (*P* < 0.05).

To assess the impact of gut bacteria, each selected strain was reintroduced to second-instar axenic *H. vigintioctopunctata* larvae. Kaplan–Meier survival analysis revealed a substantial increase in mortality in axenic larvae fed ds*ACT* and reintroduced to these bacterial isolates compared to ds*GFP*-fed controls. While the ds*ACT*-fed group exhibited a survival rate of nearly 60% at 7 days post-feeding, all bacterial strains combined with ds*ACT* led to mortality rates close to 100% after 7 days, albeit with variations in the extent of mortality among treatments. Among the tested isolated, the group treated with *E. coli* exhibited the most pronounced effects, with almost 90% mortality by day 5, followed by *Enterobacter sp*., *A. encheleia*, *E. avium*, and *P. allii* in descending order (Fig. 6A). RT-qPCR analysis confirmed a consistent downregulation of *β-Actin* gene expression across all bacterial re-introduction groups, indicating that these bacteria enhance RNAi efficacy without directly influencing the RNAi machinery (Fig. 6B).

**Fig. 7:**
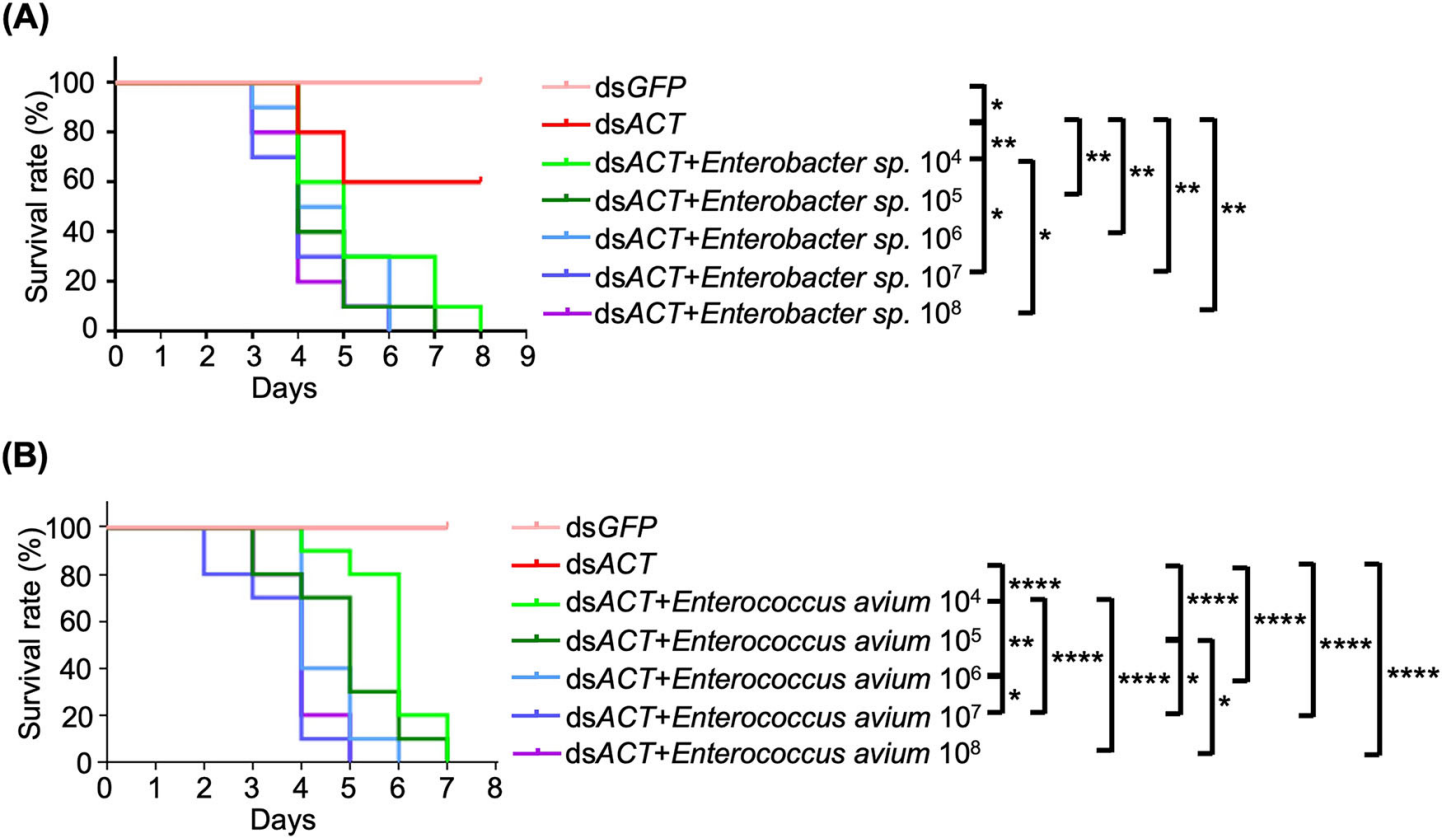
Bacterial dosage-dependent enhancement of mortality in axenic second-instar larvae with ds*ACT* feeding. Kaplan–Meier survival curves of axenic *H. vigintioctopunctata* larvae (*n* = 30) fed with ds*ACT* (4 ng/cm^2^) after the reintroduction of *Enterobacter sp*. (**A**) *or E*. avium (**B**), respectively. The log-rank test was used evalutate the significance of differences between the survival curves. ****, *P* < 0.0001; ***, *P* < 0.001; **, *P* < 0.01; *, *P* < 0.05.

The influence of bacterial density on modulating RNAi efficacy was explored by reintroducing *Enterobacter sp*. and *E. avium* at varying concentrations into axenic larvae. Survival analysis revealed a clear dose-dependent relationship, where higher bacterial densities led to significantly greater larval mortality when combined with ds*ACT* feeding (Fig. 7A, B). These findings highlight the pivotal role of bacterial density in determining the magnitude of RNAi effects, emphasizing that optimal bacterial densities are crucial to maximize RNAi-induced lethality.

### Impact of re-introduction of gut bacteria into non-axenic larvae

**Fig. 8:**
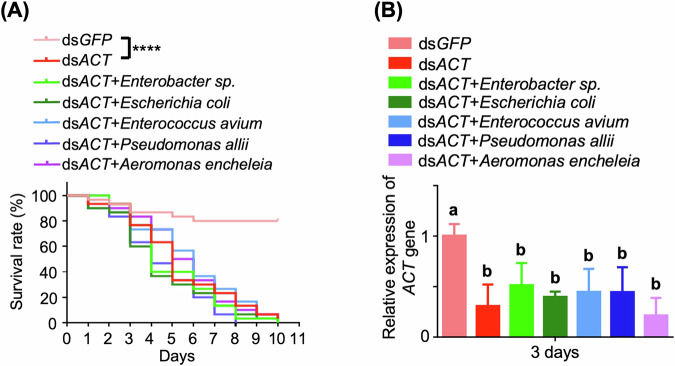
Determination of bacterial re-introduction to non-axenic second-instar larvae with ds*ACT* feeding. **A** Kaplan–Meier survival curves of *H. vigintioctopunctata* non*-*axenic larvae (*n* = 30) fed with ds*ACT* (4 ng/cm^2^) after the reintroduction of bacterial species. Bacterial density of 10^4^ cells/cm^2^ was applied to *S. nigrum* leaves. The significance of differences between the two survival curves was evaluated using the log-rank test. ****P < 0.0001. **B** Relative *β*-*Actin* gene expression in *H. vigintioctopunctata* larvae from (**A**) at 3 days post-feeding. *HvRPL13* was utilized as an internal control, with gene expression levels normalized to 1 in larvae fed with ds*GFP* leaves. Data are means ± SD (*n* = 3). Different letters above the bars indicate a significant difference, as determined by one-way ANOVA (*P* < 0.05).

The impact of bacterial re-introduction was further investigated in non-axenic larvae. Kaplan–Meier survival analysis revealed that second-instar non-axenic larvae reintroduced with gut bacterial isolates displayed significantly heightened mortality when fed with ds*ACT* compared to ds*GFP*-fed controls. However, no significant differences were observed in the groups of larvae reintroduced with bacterial strains and fed ds*ACT* compared to those solely fed ds*ACT* (Fig. 8A). RT-qPCR analysis confirmed a notable suppression of *β*-*Actin* gene expression across all treatment groups (Fig. 8B). Additional assessments were conducted to evaluate the impact on non-axenic larvae through bacterial re-introduction assays with varying bacterial dosages or different ds*ACT* concentrations. No significant differences were detected among different bacterial dosages when compared to the group fed with only ds*ACT* (Supplementary Fig. 5A). As the dosage of ds*ACT* increased, mortality also increased, yet no significant differences were observed between groups fed solely with ds*ACT* and those fed with bacterial reintroduction (Supplementary Fig. 5B).

Given that different larval developmental stages may exhibit distinct RNAi sensitivity, the impact of bacterial re-introduction using first-instar non-axenic larvae was assessed. As depicted in Supplementary Fig. 6A, the group fed with ds*ACT* demonstrated significantly increased mortality compared to the ds*GFP*-fed group, with no significant variations observed among the groups fed with ds*ACT* alone and those with each of the five different bacterial strains reintroduced. RT-qPCR analysis confirmed substantial silencing of the *β-Actin* gene across all treatment groups in comparison to the ds*GFP*-fed group (Supplementary Fig. 6B). Considering additional criteria such as larval mean weight and the area consumed by larval feeding during the bioassay, the supplementary reintroduction of gut bacterial strains to first-instar non-axenic larvae did not yield significant differences compared to those fed with ds*ACT* alone (Supplementary Fig. 6C, D).

These results indicate that the supplementary reintroduction of gut bacteria to non-axenic larvae, irrespective of varying larval developmental stages, did not impact RNAi efficacy. This implies that the level or quantity of native microbiota in non-axenic larvae is adequate to amplify the lethal effects of ds*ACT*.

### Applicability of RNAi in crop systems: transplastomic potato plants

**Fig. 9:**
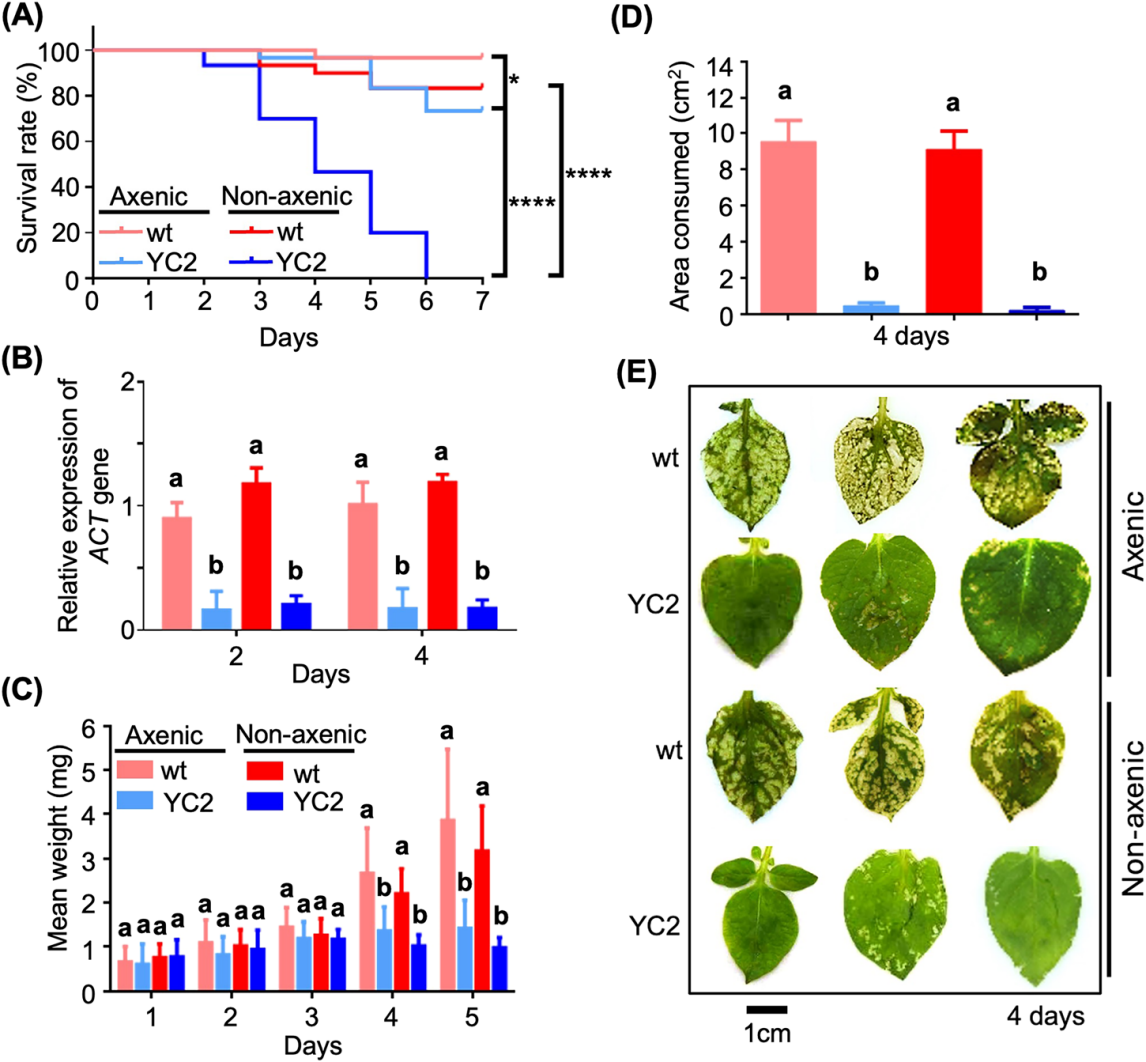
Feeding assays of second-instar *H. vigintioctopunctata* larvae using potato transplastomic plants. **A** Kaplan–Meier survival curves of second-instar *H. vigintioctopunctata* larvae fed with aseptic transplastomic potato leaves YC2 (ds*ACT*) and WT (control) to assess survival in axenic larvae and non-axenic larvae (*n* = 30). The log-rank test evaluated the significance of differences between the survival curves. Statistical significance indicated by different letters at *P* < 0.05. *P* values for each group pair are displayed on the right. **B** Relative *β*-*Actin* expression levels in *H. vigintioctopunctata* larvae from (**A**) on days 2 and 4. *HvRPL13* served as an internal control. Data are means ± SD (*n* = 3). Different letters above the bars indicate significant difference, as determined by one-way ANOVA (*P* < 0.05). **C** Mean weights of surviving *H. vigintioctopunctata* larvae. Different letters above the bars indicate significant differences between groups (*P* < 0.05, one-way ANOVA with Tukey’s multiple comparison test). **D** Comparison of feeding damage caused by *H. vigintioctopunctata* larvae after 4 days of feeding. **E** Leaf area consumed by *H. vigintioctopunctata* larvae, as shown in (**D**). Scale bar, 1 cm.

To assess the broader utility of RNAi-based pest control strategies in crop systems, transplastomic potato plants expressing ds*ACT* in plastid (termed YC2)^6^ were investigated against *H. vigintioctopunctata*. Kaplan–Meier survival analysis revealed that non-axenic larvae fed with YC2 leaves exhibited significantly increased mortality, approaching 100% at 6 days post-feeding, when compared to those fed with wild-type (WT) potato leaves (Fig. 9A). Conversely, even axenic larvae fed with YC2 demonstrated a notable difference in survival compared to those fed with WT treatments; however, the mortality of axenic larvae fed with YC2 was considerably lower, reaching nearly 30% at 6 days post-feeding, emphasizing the essential role of gut microbiota in ds*ACT* efficacy.

Consistent with the observations with *S. nigrum* (Fig. 2), RT-qPCR analysis revealed substantial suppression of *β*-*Actin* gene expression in axenic and non-axenic larvae fed with YC2 leaves in comparison to WT-fed controls, respectively (Fig. 9B). Phenotypic assessments regarding larval mean weight and the area of leaf consumed by larval feeding indicated that axenic or non-axenic larvae consuming YC2 leaves displayed significantly reduced weight and feeding damage compared to those feeding on WT leaves (Fig. 9C–E). These findings underscore the necessity of gut microbiota for the efficacy of dsRNA-expressing transplastomic potatoes in controlling *H. vigintioctopunctata* and highlight the potential for utilizing RNAi technology in diverse crop systems.

## Discussion

The results of this study underscore the pivotal role of gut microbiota in augmenting the effectiveness of RNAi against *H. vigintioctopunctata*. The findings illustrate that gut bacteria enhance RNAi impacts through mechanisms such as inducing dysbiosis, modulating host-microbiome interactions, and influencing metabolic pathways that heighten physiological stress in the host. The successful utilization of transplastomic potatoes expressing ds*ACT* highlights the potential for integrating RNAi into sustainable pest management approaches, particularly for addressing significant agricultural pests. Moreover, the observed dose-dependent effects of bacterial density and the specificity of bacterial contributions offer valuable insights for refining and optimizing RNAi strategies.

The significance of gut microbiota in RNAi-mediated pest management strategies has emerged as a critical determinant of the efficacy of these approaches^8,9^. Our results align with and extend the expanding body of knowledge indicating that gut microbial communities play a crucial role in modulating insecticidal activity through intricate host-microbe interactions. Recent investigations have emphasized the essential contribution of gut bacteria in enhancing the effectiveness of RNAi-based pest control strategies. For instance, a previous study illustrated how gut bacteria accelerate the action of *B. thuringiensis* (Bt) Cry3Bb in *P. versicolora*^15^, a model organism closely related to our study system. Additionally, studies on plastid-mediated RNA interference (PM–RNAi) targeting essential insect genes in transplastomic plants have demonstrated the influence of gut microbial dynamics on the efficacy of these approaches^9^. Consistent with our experimental observations (Fig. 2A), control larvae (mock and ds*GFP*-treated) typically initiated pupation 9–10 days post-feeding. In contrast, ds*ACT*-treated axenic larvae exhibited progressive mortality during the larval stage without reaching pupation, indicating a delayed yet eventually lethal effect. Notably, non-axenic larvae receiving ds*ACT* treatment demonstrated accelerated mortality compared to their axenic counterparts. Our study substantiates these findings, particularly highlighting that gut dysbiosis triggered by RNAi can intensify insect mortality, potentially attributable to the transition of specific bacterial populations from commensal to pathogenic states. We propose that ds*ACT*-mediated damage to the gut epithelium compromises intestinal barrier function^8,9^, facilitating bacterial translocation into the hemocoel. This breach may lead to systemic infection and account for the heightened mortality observed in non-axenic larvae.

Research focusing on *H. vigintioctopunctata* has revealed that the composition of its gut bacterial community fluctuates with diet and influences the insect’s physiological responses to RNAi^18^. Our findings align with this body of research, demonstrating that reintroducing specific bacterial strains enhances RNAi efficacy by inducing dysbiosis, thereby corroborating the findings of researchers regarding the microbial influence on dsRNA degradation and insecticidal activity^8,9^. Mechanistically, the efficacy of RNAi is, in part, governed by the roles of gut bacteria in dsRNA uptake and degradation^8,19^. Research indicates that bacterial degradation products of dsRNA can be utilized for bacterial proliferation, indirectly amplifying insect lethality. The dynamics of these intricate interactions were clearly observed in our experiments, where non-axenic insects displayed significantly higher mortality rates compared to axenic counterparts. This mortality difference was associated with gut dysbiosis (Fig. 3), characterized by distinct microbial community shifts in ds*ACT*-fed non-axenic larvae relative to other treatments. PICRUSt2 analysis (Fig. 4) revealed the potential functional consequences of this dysbiosis, showing increased abundance of metabolic pathways related to nucleotide metabolism and amino acid biosynthesis. This pattern may reflect microbial adaptation to the dsRNA-modified gut environment. However, as these are predictive functional analyses, they should be interpreted as indirect evidence of microbial community adaptation rather than direct assessments of metabolic activity.

The cellular interactions between insects and gut microbiota involve pattern recognition receptors (PRRs) such as peptidoglycan recognition proteins (PGRPs) and gram-negative binding proteins (GNBPs) that detect microbial-associated molecular patterns (MAMPs)^20–24^. Activation of these PRRs triggers signalling pathways like Toll and IMD, crucial for regulating antimicrobial peptide production and maintaining gut homeostasis. These intricate interactions play a central role in gut homeostasis and responding to microbial disruptions^25–28^. Our results suggest that ds*ACT* treatment induces dysbiosis and alters the cellular recognition of gut microbiota. Dysbiosis likely influences the composition and abundance of MAMPs, thereby impacting the activation of immune pathways, such as Toll and IMD, within gut epithelial cells. These changes in MAMP profiles may affect the sensitivity and specificity of PRRs, leading to varied downstream signalling cascades^29–32^. This reprogramming of immune responses underscores the intricate interplay between dysbiosis and host immune modulation at the cellular level. This altered cellular response may enhance the effects of RNAi by disrupting the microbial equilibrium and amplifying pathogen-like signals that trigger heightened immune activity. Subsequently, these pathways activate antimicrobial peptide production and other immune effectors, potentially intensifying the insect’s immune response and augmenting RNAi efficacy^33–35^. Additional mechanisms may also be involved, including microbe-mediated toxin production, impaired detoxification, disruption of immune homeostasis, and autoimmunity^36^. Further exploration of these specific signals could deepen our understanding of the complex interplay between microbial recognition and RNAi outcomes.

RNAi functions as a crucial antiviral defense mechanism in insects, primarily mediated by the small interfering RNA (siRNA) pathway that targets and degrades viral RNA. Viral infections can substantially alter RNAi efficacy by either saturating the RNAi machinery or producing viral suppressors of RNAi (VSRs) that inhibit pathway function^37,38^. While our study did not quantify viral loads in axenic or non-axenic insects, we hypothesize that axenic insects reared under sterile conditions are likely virus-free, whereas non-axenic populations may harbor vertically transmitted viruses. Such virome differences could potentially influence both RNAi efficacy and phenotypic outcomes. Although our bacterial reintroduction experiments demonstrate that gut microbiota modulate RNAi responses, we cannot rule out potential viral contributions to these effects. Future metagenomic studies characterizing the insect virome could elucidate viral community dynamics and their interactions with the RNAi pathway.

The advent of PM–RNAi has significantly enhanced pest control efficacy by addressing the instability concerns associated with nuclear transgenic methods. PM-RNAi involves expressing dsRNAs in plastids, enabling higher dsRNA accumulation and stability^4,6,13,14^. Our utilization of transplastomic plants expressing ds*ACT* against *H. vigintioctopunctata* showcased superior mortality rates, particularly in the presence of gut microbiota, supporting the notion that harnessing microbiota dynamics can optimize PM–RNAi efficacy further^9^.

The integration of gut microbiota dynamics into pest control strategies holds promise for revolutionizing agricultural practices^39,40^. The synergistic relationship between RNAi and gut microbiota offers an eco-friendly alternative to chemical pesticides, mitigating off-target effects and resistance development^12,41,42^. Nevertheless, challenges persist in standardizing microbial reintroductions and understanding species-specific variations in gut microbial interactions.

Future research endeavors should delve into the molecular mechanisms governing gut-microbe-mediated RNAi enhancement, with a focus on identifying key bacterial strains and their interactions with dsRNA. Field trials are essential for validating laboratory findings and assessing ecological impacts. Exploring microbiota-targeted molecular factors to amplify RNAi responses presents a promising research avenue. Additionally, further investigations should explore how dysbiosis-induced alterations in microbial recognition at the cellular level influence immune signalling and RNAi outcomes. Unravelling these pathways will provide profound insights into the intricate interplay between host immunity and microbiota during RNAi treatments. Our study underscores the pivotal role of gut microbiota in modulating RNAi efficacy by revealing how dsRNA-induced dysbiosis amplifies physiological stress and lethality in *H. vigintioctopunctata*. We demonstrate the dose-dependent impact of bacterial reintroduction and the essential contribution of microbiota in optimizing RNAi effects in the transplastomic crop system. These findings highlight the potential of microbiota-informed strategies in developing sustainable pest management approaches that integrate microbial dynamics to enhance RNAi efficacy. By capitalizing on these insights, we can advance more precise agricultural practices, paving the way for innovative solutions in pest control and crop protection.

## Methods

### Plant and insect materials

*Solanum nigrum L*. seeds underwent sterilization and vernalization at 4 °C for 24 h before germination on Murashige and Skoog (MS) medium. Seedlings were subsequently transplanted into sterile culture bottles or potting soil for cultivation in a greenhouse setting. Wild-type and ds*ACT* expressing plastid transformants of *Solanum tuberosum* cv. Désirée were propagated in sterile MS medium. Cultivation conditions included a temperature of 22 °C, light intensity of 90 μE/m²/s, and a photoperiod of 16 h light and 8 h dark^6^.

Adult *H. vigintioctopunctata* were collected in September 2022 from Nanyang, Henan Province, China (latitude 34.40°N, longitude 112.21°E). Both adult *H. vigintioctopunctata* and larvae were reared on fresh *S. nigrum* leaves within transparent rectangular plastic boxes lined with moist filter paper. The developmental cycle encompassed four stages: egg, larva, pupa, and adult. Rearing conditions were maintained at 27 ± 1 °C, with a relative humidity of 50–60% and a photoperiod of 14 h light and 10 h dark.

### Culture media

Details regarding the various culture media used in this study are outlined in Supplementary Table 3.

### Preparation of axenic *H. vigintioctopunctata* larvae

Newly laid eggs were soaked in 75% ethanol for 10 min for sterilization. After washing with sterilized water three times every three minutes and air drying, individual eggs were transferred onto LB solid medium. Successful removal of gut bacteria was verified by colony-forming unit (CFU) assay on an LB plate and PCR amplification using conserved primers targeting the 16S *rRNA* gene of gut bacteria (Fig. 1A, B). Upon hatching, the larvae were carefully transferred to aseptic detached *S. nigrum* leaves within a controlled sterile environment set at 28 °C, 60% relative humidity, and 16-h light/8-h dark photoperiod. *S. nigrum* plants, used as the food source for the larvae, were cultivated under aseptic conditions on MS medium.

### In vitro dsRNA synthesis and feeding assay

Gene fragments of *β*-*Actin* and *GFP* were PCR amplified using gene-specific primer pairs (Supplementary Table 4) and subsequently served as templates for the synthesis of dsRNA (ds*ACT* and ds*GFP*) using the T7 RiboMAX^TM^ Express RNAi system (Promega, USA) following the manufacturer’s instructions. The integrity of the synthesized dsRNAs was assessed through electrophoresis on 1.5% agarose gels, and the quantities of dsRNA were determined using a spectrophotometer (Nano-Drop 2000, Thermo Scientific, USA).

For the insect larvae feeding assay, first or second-instar larvae (*n* = 30) were provided with sterile *S. nigrum* leaves coated with either sterile water, ds*GFP*, or ds*ACT* at concentrations of 4 ng/cm². Daily assessments were conducted to record mortality rates, larval weight, and leaf consumption. Leaf-feeding behaviours were documented through photographic recordings. Larvae from each group were sampled at 2- and 4-days post-feeding for subsequent gene expression analysis.

### Gene expression analysis

Quantitative real-time PCR (qPCR) was performed to assess the relative expression levels of the *β-Actin* gene in *H. vigintioctopunctata*, the target of ds*ACT*. Two different H. vigintioctopunctata genes, ribosomal protein L13 gene *RPL13(HvRPL13)* and glyceraldehyde 3-phosphate dehydrogenase gene *GAPDH (HvGAPDH)*, were used as reference genes for normalization to deepen the target gene expression reliability^43^. Total RNA extraction from larval samples was carried out using the TransZol Total RNA Extraction Kit (TransGen, Beijing, China) following the manufacturer’s protocol. Larval samples were collected from different conditions, including larvae fed on leaves coated with dsRNA (ds*ACT* or ds*GFP*) or sterile water. For each treatment group, total RNA was extracted from three biological replicates, with each replicate comprising pooled larvae (*n* = 10). For reverse transcription-PCR (RT-PCR), cDNA was synthesized from total RNA (1 μg) using Hifair^®^ II 1st Strand cDNA Synthesis Kit (Yeasen, Shanghai, China) according to the manufacturer’s instructions. For qPCR, PCR was performed for 45 cycles using 1 μL of cDNA as a template. Hieff^®^ qPCR SyBR Green Master Mix (Yeasen, Shanghai, China) and CFX Connect Real-Time PCR Detection System (Bio-Rad, Hercules, CA, USA) were used following the manufacturer’s instructions. The relative expression levels of *β-Actin* gene were calculated using the 2^ − ΔΔCT method, where the cycle threshold (CT) values for the target gene (*β-Actin*) and the reference gene (*RPL13*) were used to determine fold changes in gene expression relative to control samples. Each biological replicate included three technical replicates to ensure data reliability. Statistical analysis of all RT-qPCR data was conducted using GraphPad Prism 9 software. Statistical comparisons between treatment groups were performed via one-way ANOVA followed by Tukey’s post hoc test, with significance set at *P* < 0.05.

### Analysis of gut microbiota

To investigate the composition and diversity of gut microbiota in *H. vigintioctopunctata*, second-instar larvae were used after feeding on sterile *S. nigrum* leaves coated with ds*ACT*, ds*GFP*, or sterile water for three days. To minimize external microbial contamination, larvae were surface-sterilized by immersion in 75% ethanol for 2–3 min, followed by triple rinsing with sterile water. Under sterile conditions, guts were carefully dissected by making a small incision at the larval posterior with a sterile scalpel. The gut tissues were extracted using sterilized forceps and pooled into sterile, RNase-free 2.0 mL centrifuge tubes. For each treatment group, six biological replicates were prepared, with each replicate consisting of guts pooled from five larvae. The samples were flash-frozen in liquid nitrogen post-dissection and stored at −80 °C for subsequent analysis.

Genomic DNA from the gut tissues was extracted using the Biomarker Blood/Cell/Tissue DNA Kit (Biomarker, China) according to the manufacturer’s protocol. DNA integrity and concentration were evaluated using a Nano-Drop spectrophotometer and agarose gel electrophoresis. The V3–V4 hypervariable regions of the bacterial 16S *rRNA* gene were PCR amplified with the universal primer pair 338 F and 806 R. Each 25 μL PCR reaction contained 12.5 μL of Phusion High-Fidelity PCR Master Mix, 0.2 μM of each primer, and 10 ng of template DNA. The PCR conditions included an initial denaturation at 98 °C for 30 s, followed by 30 cycles of 98°C for 10 s, 55 °C for 30 s, and 72 °C for 30 s, with a final extension at 72 °C for 5 min. PCR products were purified with the E.Z.N.A.® Gel Extraction Kit (Omega Bio-Tek, USA) and quantified using a Qubit Fluorometer (Thermo Fisher Scientific, USA).

Amplicon libraries were prepared with the NEBNext® Ultra™ DNA Library Prep Kit for Illumina sequencing and sequenced on an Illumina MiSeq platform (Majorbio, Shanghai), generating 300 bp paired-end reads. Raw reads were processed to ensure high data quality. First, *fastp* (version 0.19.6) was used to trim low-quality bases (*Q* < 20) from the ends of reads, and reads shorter than 50 bp or containing ambiguous bases (*N*) were discarded. Paired-end reads were then merged using *FLASH* (version 1.2.11) with a minimum overlap of 10 bp and a maximum mismatch ratio of 0.2. Barcodes and primer sequences were used to assign reads to samples, allowing no mismatches in barcodes and up to two mismatches in primers. Quality-filtered reads were processed using the DADA2 plugin in QIIME2 (version 2020.6) to denoise sequences, generating high-resolution amplicon sequence variants (ASVs). Taxonomic classification of ASVs was performed using a Naive Bayes classifier trained on the SILVA 16S *rRNA* gene database (version 138). Sequences corresponding to chloroplasts and mitochondria were excluded from the dataset. Detailed sequencing data obtained from MiSeq was shown in Supplementary Table 5.

To account for differences in sequencing depth, all samples were rarefied to 20,000 sequences per sample. The Good’s coverage estimator confirmed that this sequencing depth captured over 99% of microbial diversity. Alpha diversity metrics, including the Chao1 richness estimator and Shannon diversity index, were calculated using the *mothur* software. Intergroup differences were assessed using the Wilcoxon rank-sum test. Beta diversity was analysed through Principal Coordinate Analysis (PCoA) based on Bray-Curtis dissimilarity distances, and statistical significance was assessed with Permutational Multivariate Analysis of Variance (PERMANOVA). Functional predictions were made with PICRUSt2 (version 2.2.0) and mapped to KEGG ortholog pathways (Supplementary Table 1). Data processing, statistical analyses, and visualization were conducted on the Majorbio Cloud Platform (https://cloud.majorbio.com). This comprehensive workflow facilitated a detailed exploration of gut microbial community structure, diversity, and functional contributions under dsRNA treatments.

### Isolation of gut bacteria

Gut bacteria were isolated by plating homogenized gut samples on selective media. Bacterial colonies were purified and identified through 16S rRNA sequencing (Supplementary Table 2). Larvae underwent surface sterilization by brief immersion in 75% ethanol for 15 s to eliminate external contaminants, followed by triple rinsing with sterile water. Under aseptic conditions, larvae were dissected using sterile forceps and scalpels to extract their guts. Approximately 15 guts were pooled into a sterile 2 mL microcentrifuge tube with 100 µL of sterile phosphate-buffered saline (PBS) and small stainless-steel beads for tissue homogenization. The pooled gut tissues were homogenized using a bead-beating process to release bacteria into the suspension. Serial dilutions were made in sterile PBS to achieve dilution factors of 10^2^, 10^3^, 10^4^, and 10^5^. Each dilution was spread onto the surface of five different agar media types to support diverse bacterial growth, including LB (Luria-Bertani), Nutrient Agar (NA), Salmonella-Shigella (SS) Agar, Enterococcus Agar, and Pseudomonas CFC Selective Agar (Supplementary Table 2). Plates were incubated at 28 °C for 48–72 h. After incubation, bacterial colonies with distinct morphologies were chosen from the plates based on variations in size, shape, and colour. These colonies were streaked onto fresh plates of the same medium for purity confirmation. Purification steps were repeated as needed until isolates displayed uniform morphology. The purified isolates underwent molecular identification. Genomic DNA was extracted from each isolate using the Biomarker Blood/Cell/Tissue DNA Kit following to the manufacturer’s protocol. The 16S *rRNA* gene was PCR amplified using universal primers 27 F and 1492 R. Each PCR reaction consisted of 25 μL, containing 12.5 μL of 2× Phusion High-Fidelity PCR Master Mix, 0.2 μM of each primer, and 10 ng of template DNA. The thermal cycling conditions were as follows: initial denaturation at 98 °C for 30 s, followed by 30 cycles of 98 °C for 10 s, 55 °C for 30 s, and 72 °C for 1 min, with a final extension at 72 °C for 5 min. PCR products were purified using the E.Z.N.A.® Gel Extraction Kit and sequenced through Sanger sequencing. The resulting sequences were compared against the NCBI GenBank database for bacterial species identification (Supplementary Table 2).

This method ensured comprehensive isolation and identification of a diverse range of gut bacteria, offering an in-depth profile of the gut microbiota linked with *H. vigintioctopunctata*. The isolated bacteria were subsequently employed for reintroduction experiments and further functional analyses.

### Re-introduction of gut bacteria to larvae

To assess how gut bacteria influence the physiology and susceptibility of *H. vigintioctopunctata* larvae, bacteria isolated from laboratory-reared larvae were reintroduced to both axenic and non-axenic larvae. The re-introduction process involved preparing bacterial suspensions, applying them to larval feeding substrates, and monitoring subsequent impacts on the larvae. Bacteria isolated during the gut microbiota study were cultured overnight in LB liquid medium at 28 °C with shaking at 220 rpm. The bacterial cultures were pelleted by centrifugation at 7000 rpm for 10 min at 4 °C, washed thrice with sterile water, and resuspended in sterile water. The bacterial suspension was adjusted to the desired concentration for the experiment, with the final concentration modified based on the experimental condition. For re-introduction, the bacterial suspension was combined with ds*ACT* at a final concentration of 4 ng/µL. For axenic larvae, sterile 2 × 2 cm *S. nigrum* leaves were prepared. The bacterial suspension (10 µL/cm²) was evenly spread on the leaves along with ds*ACT* (4 ng/cm²). These treated leaves were air-dried under sterile conditions for suspension adherence. Groups of second-instar sterile larvae (*n* = 30 per treatment) were placed in sterile Petri dishes lined with moistened sterile filter paper and provided with the treated leaves as their exclusive food source. Control groups were fed leaves coated with ds*GFP* and no bacteria. Non-axenic larvae were treated similarly, except without sterilization, to maintain the natural microbial community. The bacterial suspension (10 µL/cm²) and ds*ACT* (4 ng/cm²) were applied to the leaves, which were then air-dried. Second-instar non-axenic larvae (*n* = 30 per treatment) were placed in Petri dishes with moistened filter paper and fed the treated leaves. Control groups were provided with leaves treated with ds*GFP* but without additional bacteria. Treated leaves were switched daily with fresh leaves to ensure consistent treatment, prevent desiccation or contamination. Larval mortality, body weight, and leaf consumption were recorded daily. Dead larvae were promptly removed and documented. At 3 days post-feeding, larvae from each treatment group were sampled for RT-qPCR analysis to evaluate gene expression changes due to bacterial re-introduction. Mortality rates and larval weight were statistically analysed using one-way ANOVA followed by Tukey’s post hoc test to determine significant differences between treatment groups (*P* < 0.05).

## Supplementary information

Supplementary information

## Data Availability

The raw sequence reads from this study are available from the NCBI Sequence Read Archive (SRA) under the accession number PRJNA1210164. The data supporting the findings of the study are available from the corresponding author upon request.
