# Supplementary material for: The essential role of gut microbiota in dsRNA-mediated pest control of the phytophagous ladybird beetle, Henosepilachna vigintioctopunctata
Source: NPJ Biofilms Microbiomes. 2025 Jul 22;11:141. doi: 10.1038/s41522-025-00767-x (PMC12280057; doi:10.1038/s41522-025-00767-x)
Supplement: Supplementary file 1 — Supplementary information [file 41522_2025_767_MOESM1_ESM.pdf]

Supplementary information to

The essential role of gut microbiota in dsRNA-mediated pest control of the phytophagous ladybird beetle, *Henosepilachna vigintioctopunctata*

Yujie Huang[1#], Jinman Huang[1,2#], Dae Sung Kim[1*], Jiang Zhang[1, 2*]

[1] School of Life Sciences, Hubei University, Hubei Hongshan Labortory, Wuhan 430062, China

[2] Shenzhen Branch, Guangdong Laboratory of Lingnan Modern Agriculture, Key Laboratory of Synthetic Biology, Ministry of Agriculture and Rural Affairs, Agricultural Genomics Institute at Shenzhen, Chinese Academy of Agricultural Sciences, Shenzhen, 518000, China

[#]These authors contribute equally to this work.

Corresponding author: Dae Sung Kim (daesungkim@hubu.edu.cn); Jiang Zhang (zhangjiang@hubu.edu.cn)

This PDF file contains
Supplementary Table 1-5
Supplementary Figure 1-6

Supplementary Table 1. Indicated EC numbers in Fig. 4. and its enzymatic function.

EC number	Function
4.2.1.20	tryptophan synthase
3.1.26.4	ribonuclease H
6.3.5.5	carbamoyl-phosphate synthase
2.7.11.1	non-specific serine/threonine protein kinase
1.2.1.12	glyceraldehyde-3-phosphate dehydrogenase
2.8.1.7	cysteine desulfurase
1.20.4.1	arsenate reductase
3.1.11.5	exodeoxyribonuclease V
3.6.3.21	ABC-type polar-amino-acid transporte
4.2.1.3	aconitate hydratase
5.99.1.3	DNA topoisomerase
2.5.1.47	cysteine synthase
2.3.1.128	ribosomal-protein-alanine N-acetyltransferase
6.3.5.6	asparaginyl-tRNA synthase
6.3.5.7	glutaminyl-tRNA synthase
4.3.3.7	4-hydroxy-tetrahydrodipicolinate synthase
3.1.4.46	glycerophosphodiester phosphodiesterase
1.2.1.16	succinate-semialdehyde dehydrogenase [NAD(P)+]
1.7.99.4	nitrate reductase
4.2.99.18	DNA-(apurinic or apyrimidinic site) lyase
1.15.1.1	superoxide dismutase
3.1.21.3	type I site-specific deoxyribonuclease
1.2.4.1	pyruvate dehydrogenase
2.2.1.1	transketolase
3.2.1.86	6-phospho-β-glucosidase
3.6.3.14	H+-transporting two-sector ATPase
4.2.1.17	enoyl-CoA hydratase
1.17.4.1	ribonucleoside-diphosphate reductase
3.6.3.12	K+-transporting ATPase
3.6.3.17	monosaccharide-transporting ATPase
3.4.16.4	serine-type D-Ala-D-Ala carboxypeptidase
1.3.5.4	fumarate reductase
3.6.3.34	iron-chelate-transporting ATPase
2.1.1.72	site-specific DNA-methyltransferase
3.6.1.27	undecaprenyl-diphosphate phosphatase
2.3.1.9	acetyl-CoA C-acetyltransferase
2.5.1.18	glutathione transferase
1.1.1.1	alcohol dehydrogenase
1.11.1.15	peroxiredoxin
6.4.1.2	acetyl-CoA carboxylase
2.7.1.69	protein-Npi-phosphohistidine-sugar phosphotransferase
2.7.7.6	DNA-directed RNA polymerase
1.1.1.100	3-oxoacyl-[acyl-carrier-protein] reductase
2.2.1.6	acetolactate synthase
3.6.4.13	RNA 5'-3' helicase
5.2.1.8	peptidylprolyl isomerase
1.6.5.3	Oxidoreductases
2.7.13.3	histidine kinase
3.6.4.12	DNA 5'-3' helicase
2.7.7.7	DNA-directed DNA polymerase

Supplementary Table 2. Isolated bacterial species from *H. vigintioctopunctata*. Bold highlighted species were used for bacterial re-introduction assay in this study.

Strain No.	Taxon	Similarity	Family	Medium
**LB1**	***Enterobacter sp.***	99%	Enterobacteriaceae	LB
LB3	*Citrobacter freundii*	99%	Enterobacteriaceae	
LB5	*Klebsiella sp.*	99%	Enterobacteriaceae	
**NA3**	***Enterococcus avium***	99%	Enterococcaceae	NA
**NA4**	***Pseudomonas allii***	99%	Pseudomonas	
**NA5**	***Aeromonas encheleia***	99%	Aeromonadaceae	
**NA9**	***Escherichia coli***	98%	Enterobacteriaceae	
SS4	*Klebsiella aerogenes*	99%	Enterobacteriaceae	SS-Agar
J3	*Citrobacter braakii*	99%	Enterobacteriaceae	Pseudomonas CFC Selective Agar
J5	*Pseudomonas sp.*	99%	Pseudomonas	
J8	*Enterobacter hormaechei*	99%	Enterobacteriaceae	
C1	*Enterococcus faecalis*	98%	Enterococcaceae	Enterococcus Agar

Supplementary Table 3. Media used in this study.

Medium	Composition
MS (Murashige & Skoog)	4.4 g/L MS elements, 30 g/L sucrose, 5.4–5.6 g/L agar, pH adjusted to 5.8 with KOH
LB (Luria-Bertani)	5 g/L yeast extract, 5 g/L NaCl, 10 g/L peptone, and 15–20 g/L agar for solid media
NA (Nutrient Agar)	3 g/L beef extract, 10 g/L peptone, 5 g/L NaCl, 15 g/L agar, pH adjusted to 7.3
Enterococcus Agar	Various components including 3 g/L beef extract and 17 g/L casein peptone, pH adjusted to 7.1
Salmonella-Shigella Agar	Contains selective components for *Salmonella* and *Shigella* , pH adjusted to 7.0
Pseudomonas CFC Selective	Selective medium for *Pseudomonas* spp., pH adjusted to 7.1

Supplementary Table 4. Primers used in this study.

Oligo name	Sequence (5'-3')	Application
338F	ACTCCTACGGGAGGCAGCA	PCR of V3 and V4 hypervariable regions of eubacterial 16S *rRNA* gene
806R	GGACTACHVGGGTWTCTAAT	
16s-27F	AGAGTTTGATCCTGGCTCAG	PCR of eubacterial 16S *rRNA* gene
16s-1492R	TACGGYTACCTTGTTACGACTT	
GFP-F	AGTGGAGAGGGTGAAGGTGA	PCR of 200 bp of *GFP* DNA
GFP-R	CTCTCCTGCACGTATCCCTC	
ACT-F	AGGCTAACAGGGAAAAGATGAC	PCR of 200 bp fragment of *ACT* DNA
ACT-R	GTCCAAACGGAGGATGGC	
T7-GFP-F	TAATACGACTCACTATAGGAGTGGAGAGGGTGAAGGTGATGCAACATACGGAA	*In vitro* synthesis of ds*GFP*
T7-GFP-R	TAATACGACTCACTATAGGCTCTCCTGCACGTATCCCTCAGGCATGGCGCTCT	
T7-ACT-F	TAATACGACTCACTATAGGAGGCTAACAGGGAAAAGATGACCCAAATCATGTTTGAAAC	*In vitro* synthesis of ds*ACT*
T7-ACT-R	TAATACGACTCACTATAGGGTCCAAACGGAGGATGGCATGGGGGAGAGCGTAA	
HvRPL13QRT F	AGCATCCTTCGCTCGTTTAG	qRT-PCR for the internal control of *H. vigintioctopunctata*
HvRPL13QRT R	TTCGACAACCTGCCATTAGG	
Hvactin QRT F	GTGGGTATCCACGAAACCGT	qRT-PCR for *ACT* expression of *H. vigintioctopunctata*
Hvactin QRT R	ATTTCCTTTCTGGGGGAGCG	

Supplementary Table 5. Obtained sequencing data of the Illumina MiSeq from each sample.

Sample	Clean reads	Clean base (bp)	Mean length (bp)	Min length (bp)	Max length (bp)	ASVs number
H_2O_1	45285	22639008	426.796772	277	431	143
H_2O _2	47173	24024204	426.93757	398	432	140
H_2O _3	46140	23613799	427.600301	277	431	123
H_2O _4	47888	24981962	425.870033	324	432	123
H_2O _5	45165	23131423	426.99173	403	431	124
H_2O _6	45343	24150376	426.798197	263	431	104
ds*GFP*_1	41790	22066883	428.258641	403	435	113
ds*GFP*_2	41723	23535608	427.213302	324	434	121
ds*GFP*_3	59136	32103105	427.260937	324	432	135
ds*GFP*_4	46841	23834141	426.203301	262	431	117
ds*GFP*_5	49549	25934035	426.336265	371	434	112
ds*GFP*_6	50999	26767229	427.728172	276	493	110
ds*ACT*_1	43774	24510662	427.618451	222	431	116
ds*ACT*_2	46339	25130644	428.192946	403	431	76
ds*ACT*_3	36692	24138665	428.362673	261	431	99
ds*ACT*_4	43582	26557513	428.685784	234	469	88
ds*ACT*_5	45123	24009665	426.133947	277	442	117
ds*ACT*_6	40392	22870740	426.613318	277	432	107

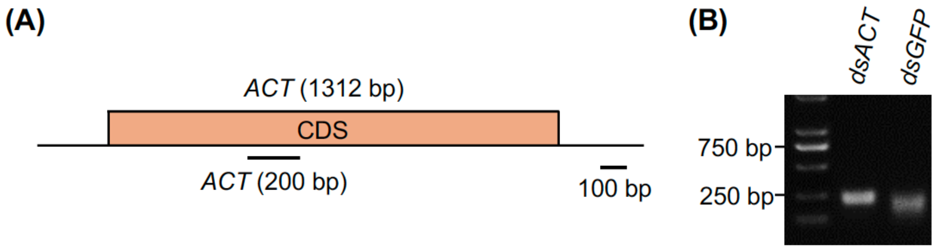

Supplementary Figure 1. *In vitro* synthesis of dsRNAs. (**A**) Target gene scheme of *H. vigintioctopunctata β-Actin* gene. CDS: coding sequence. The underlined 200 bp region was designed for the synthesis of ds*ACT*. (**B**) *In vitro* synthesis of dsRNAs, ds*ACT*, and ds*GFP* (control).

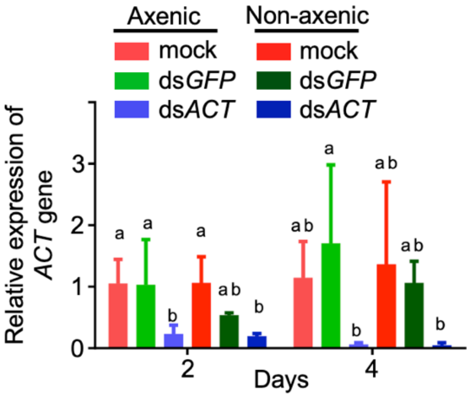

Supplementary Figure 2. Relative expression levels of *ACT* gene in *H. vigintioctopunctata* larvae normalized to *HvGAPDH*. Gene expression in *H. vigintioctopunctata* larvae fed with H_2O-painted leaves was set to 1. *HvGAPDH* served as the internal control. Data are means ± SD ($n = 3$). Different letters above the bars indicate significant differences, as determined using one-way ANOVA ($P < 0.05$).

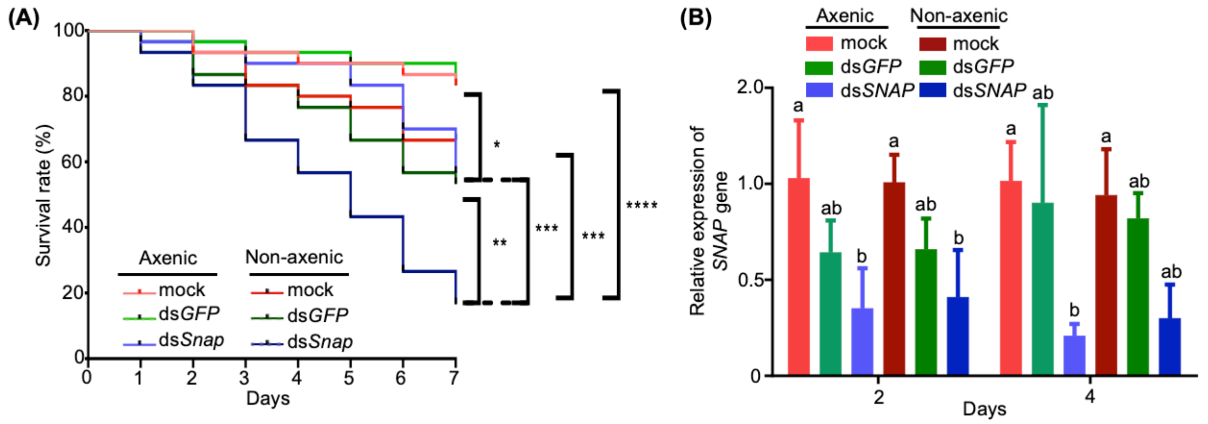

Supplementary Figure 3. Feeding assays of *H. vigintioctopunctata* with *in vitro*-synthesized ds*SNAP*. (A) Kaplan–Meier survival curves of second-instar *H. vigintioctopunctata* larvae fed with *S. nigrum* leaves painted with 4 ng/cm^2 dsRNA (ds*SNAP* or ds*GFP*) or sterilized water. Both axenic and non-axenic larvae were tested (n = 30 per group). Sterilized water or ds*GFP* served as controls. A log-rank test was used to assess the significance of the differences between the two survival curves. The statistical significance is denoted by the asterisk (*, $P < 0.05$; **, $P < 0.005$; ***, $P < 0.0005$; ****, $P < 0.0001$). (B) Relative expression levels of the *SNAP* gene in larvae from (A) on days 2 and 4. Gene expression levels in the *H. vigintioctopunctata* larvae fed with H_2O-painted leaves were set as 1, with *HvRPL13* as the internal control. Data are means ± SD (n = 3). Different letters above the bars indicate significant differences, as determined using one-way ANOVA ($P < 0.05$).

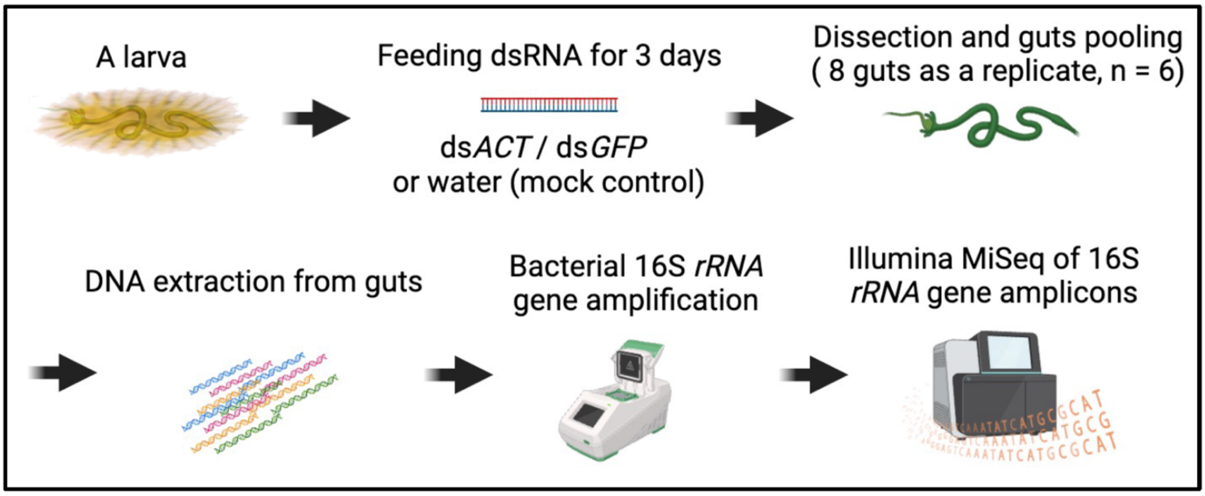

Supplementary Figure 4. Scheme of the *16S rRNA* gene sequencing process for analyzing the gut microbiota of *H. vigintioctopunctata***.**

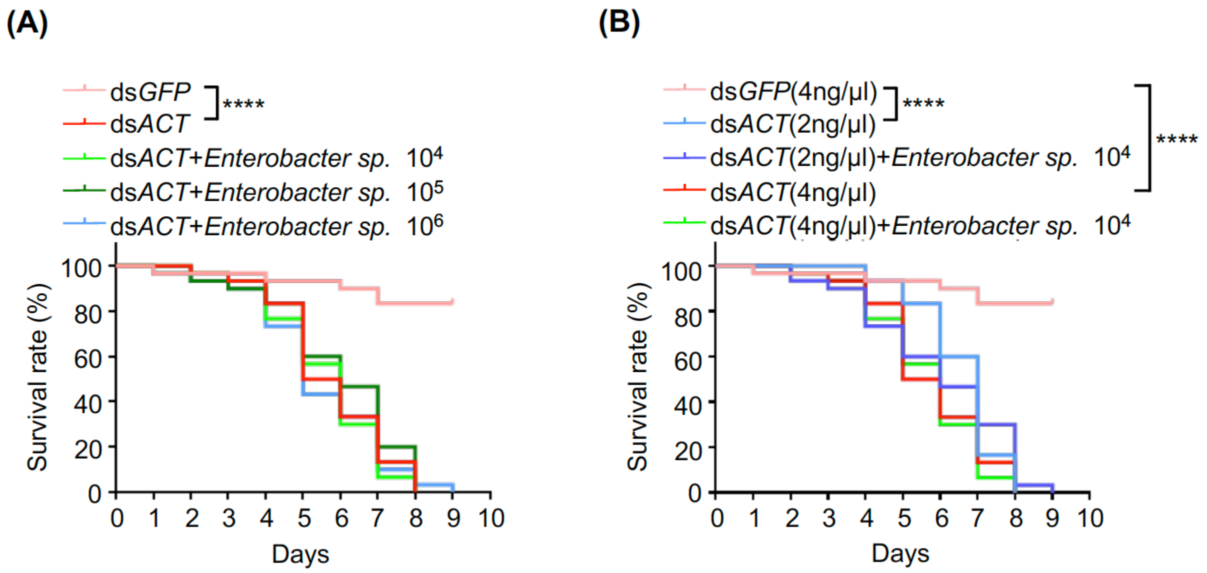

Supplementary Figure 5. Bacterial re-introduction effect on second-instar non-axenic larvae. (**A**) Kaplan–Meier survival curves of *H. vigintioctopunctata* non-axenic larvae (n = 30) fed with ds*ACT* (4 ng/cm^2) after the re-introduction of *Enterobacter sp*. with different bacterial dosages (10$^{4,\ 5\ \text{or}\ 6}$ cells/cm^2) onto *S. nigrum* leaves. (**B**) Kaplan–Meier survival curves of *H. vigintioctopunctata* non-axenic larvae (n = 30) fed with different ds*ACT* concentrations (2 or 4 ng/cm^2) after the re-introduction of *Enterobacter sp.* with 10^4 cells/cm^2 onto *S. nigrum* leaves.

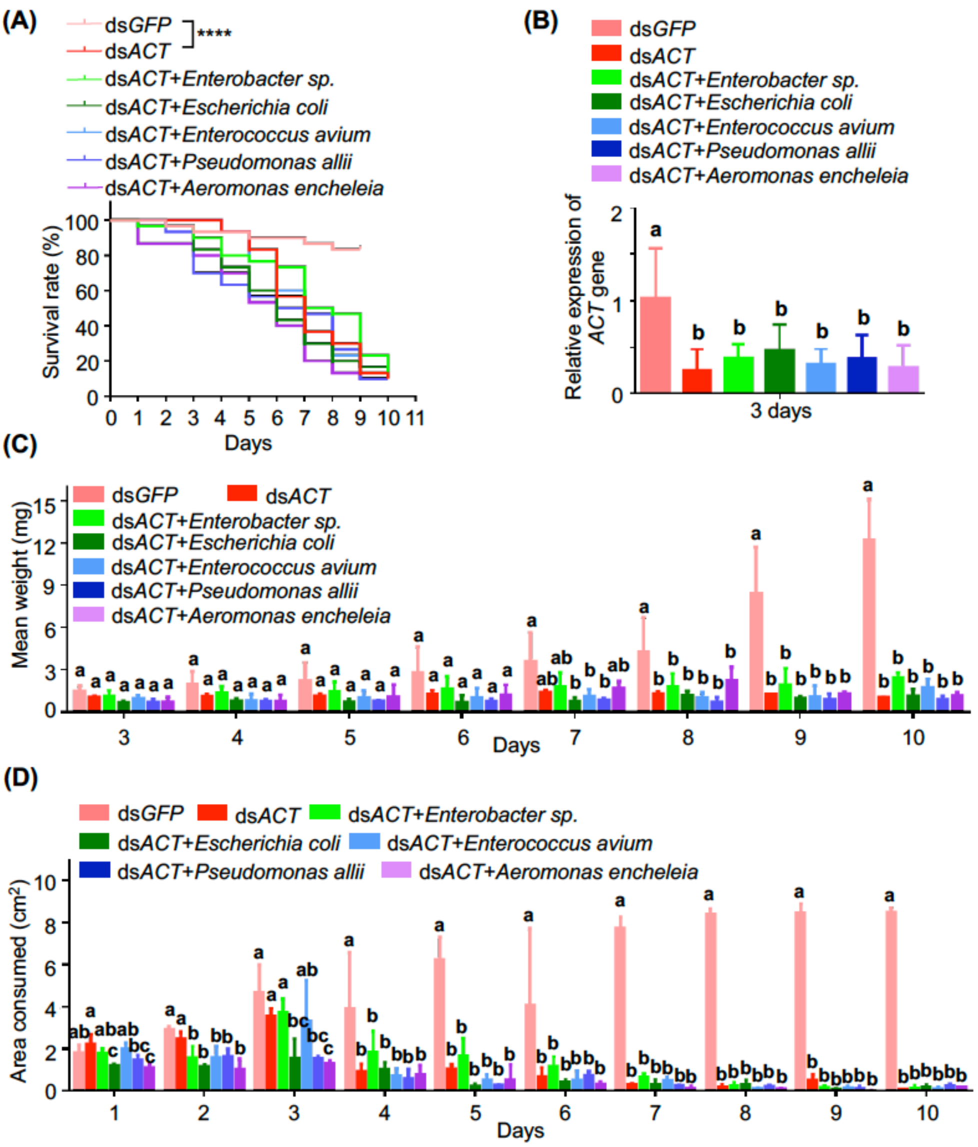

Supplementary Figure 6. Determination of bacterial re-introduction to non-axenic first-instar larvae with ds*ACT* feeding. (**A**) Kaplan–Meier survival curves of *H. vigintioctopunctata* non-axenic larvae ($n = 30$) fed with five bacterial species. Each at a bacterial density of 10^4 cells/cm^2 were applied to *S. nigrum* leaves. (**B**) Relative *β-Actin* gene expression levels in *H. vigintioctopunctata* larvae as shown in (A) on day 3. Gene expression levels in *H. vigintioctopunctata* larvae fed with ds*GFP* leaves were normalized to 1. Data are means ± SD ($n = 3$). Different letters above the bars indicate a significant difference, as determined by one-way ANOVA ($P < 0.05$). (**C**) Mean weights of the surviving *H. vigintioctopunctata* non-axenic larvae. Different letters above the bars indicate significant differences between the groups ($P < 0.05$, one-way ANOVA with Tukey's multiple comparison test). (**D**) Leaf area consumed by *H. vigintioctopunctata* larvae. Different letters above the bars indicate significant differences between the groups ($P < 0.05$, one-way ANOVA with Tukey's multiple comparison test).

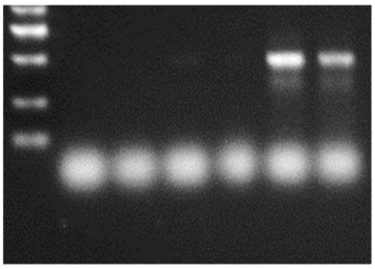

Uncropped and unprocessed scan of gel in Fig. 1 (A).
